# Emergence of Extreme Paw Accelerations During Cat Paw Shaking: Interactions of Spinal Central Pattern Generator, Hindlimb Mechanics and Muscle Length-Depended Feedback

**DOI:** 10.3389/fnint.2022.810139

**Published:** 2022-03-30

**Authors:** Boris I. Prilutsky, Jessica Parker, Gennady S. Cymbalyuk, Alexander N. Klishko

**Affiliations:** ^1^Laboratory of Biomechanics and Motor Control, School of Biological Sciences, Georgia Institute of Technology, Atlanta, GA, United States; ^2^Dynamical Neuroscience Laboratory, Neuroscience Institute, Georgia State University, Atlanta, GA, United States

**Keywords:** central pattern generator, inter-segmental energy transfer, cat paw-shake response, EMG activity, whip-like motion, neuromechanical modeling, AnimatLab

## Abstract

Cat paw shaking is a spinal reflex for removing an irritating stimulus from paw by developing extremely high paw accelerations. Previous studies of paw shaking revealed a proximal-to-distal gradient of hindlimb segmental velocities/accelerations, as well as complex inter-joint coordination: passive motion-dependent interaction moments acting on distal segments are opposed by distal muscle moments. However, mechanisms of developing extreme paw accelerations during paw shaking remain unknown. We hypothesized that paw-shaking mechanics and muscle activity might correspond to a whip-like mechanism of energy generation and transfer along the hindlimb. We first demonstrated in experiments with five intact, adult, female cats that during paw shaking, energy generated by proximal muscle moments was transmitted to distal segments by joint forces. This energy transfer was mostly responsible for the segmental velocity/acceleration proximal-to-distal gradient. Distal muscle moments mostly absorbed energy of the distal segments. We then developed a neuromechanical model of hindlimb paw shaking comprised a half-center CPG, activating hip flexors and extensors, and passive viscoelastic distal muscles that produced length/velocity-depended force. Simulations reproduced whip-like mechanisms found experimentally: the proximal-to-distal velocity/acceleration gradient, energy transfer by joint forces and energy absorption by distal muscle moments, as well as atypical co-activation of ankle and hip flexors with knee extensors. Manipulating model parameters, including reversal of segmental inertia distal-to-proximal gradient, demonstrated important inertia contribution to developing the segmental velocity/acceleration proximal-to-distal gradient. We concluded that extreme paw accelerations during paw shaking result from interactions between a spinal CPG, hindlimb segmental inertia, and muscle length/velocity-depended feedback that tunes limb viscoelastic properties.

## Introduction

Cats produce fast paw oscillations to remove water or adhesive tape on the paw (Prochazka et al., 1977, 1989; Smith et al., 1980; Abraham and Loeb, 1985; Pearson and Rossignol, 1991; Hodson-Tole et al., 2012; Mehta and Prilutsky, 2014). This is a spinal reflex, paw-shake response (Koshland and Smith, 1989; Pearson and Rossignol, 1991), aimed to remove an irritating light object stuck to the paw or foot. Previous studies have demonstrated that cats during this motor behavior develop extremely high paw velocities and accelerations, the latter often exceeding gravitational acceleration by more than 10 times (Hoy et al., 1985). There is a proximal-to-distal gradient of segmental and joint angular velocities and accelerations, with foot velocities and accelerations exceeding those of the shank and thigh by several times (Hoy et al., 1985; Hoy and Zernicke, 1986). Previous studies have also documented complex inter-joint coordination and atypical muscle synergies during paw shaking, which are dramatically different from kinematic and EMG activity patterns observed during locomotion. For example, although most of flexor and extensor hindlimb muscles demonstrate reciprocal EMG activity, as in locomotion, there is atypical co-activation between ankle flexor tibialis anterior and knee extensors vasti (Smith et al., 1985). Distal muscle moments at the ankle and knee act to counteract passive motion-depended interaction moments applied to the foot and shank (Hoy et al., 1985; Hoy and Zernicke, 1986). Thus, ankle and knee muscles are active mostly during their lengthening (Hoy et al., 1985; Fowler et al., 1988; Prochazka et al., 1989; Mehta and Prilutsky, 2014) and therefore primarily absorb energy of the foot and shank. The above description suggests that energy increase of distal segments (and their velocity and acceleration) is provided by passive interaction motion-depended forces and moments acting on the distal segments. Similar increases in angular velocity of distal limb segments have been observed in fast motor actions such as passive knee extensions during the swing phase of locomotion and kicking or throwing a ball (Bernstein, 1940; Phillips et al., 1983; Martin and Cavanagh, 1990; Wisleder et al., 1990; Putnam, 1991; Hirashima et al., 2003). The mechanism of energy transfer from proximal to distal limb segments and enhancement of velocity and acceleration of distal segments is called a whip-like mechanism, and inverse dynamics analysis has been used to quantify it (Robertson and Winter, 1980; Aleshinsky, 1986; Putnam, 1991). In whip-like motion, mechanical energy is mostly generated by muscle moments at proximal joints of the limb and is transferred to distal segments primarily by the action of joint forces that do not generate energy for the motion by themselves. Muscle moments of the distal joints primarily decelerate distal segments at the end of motion range to prevent joint overextension by absorbing energy of the distal segments (Robertson and Winter, 1980; Chapman and Caldwell, 1983; Putnam, 1991).

To achieve the proximal-to-distal gradient of segmental angular velocities and accelerations during whip-like movements, the nervous system needs to precisely regulate activities of multiple muscles and inter-joint coordination. Neural control of paw shaking is still poorly understood. Although it has been established that paw-shake-like rhythmic activity of hindlimb flexor and extensor motoneurons can be generated by spinal interneuronal networks, called central pattern generators (CPG), without motion-depended sensory feedback (Pearson and Rossignol, 1991) and that the activity of spindle group Ia afferents from muscles crossing the ankle (triceps surae) and knee joints (hamstrings) is extremely high during paw shaking (Prochazka et al., 1989), it is not known how central and feedback neural mechanisms interact to produce highly coordinated paw shaking. Previously, we proposed a neuromechanical model of cat hindlimbs controlled by a simple CPG (Parker et al., 2018), generating rhythmic reciprocal inputs to flexor and extensor motoneurons, and by motion-dependent sensory feedback, modulating the CPG and motoneuronal activity (Bondy et al., 2016). This model reproduced basic paw-shake kinematics and muscle activity patterns (i.e., reciprocal activation of hip and ankle flexors and extensors and atypical co-activation of knee extensors and ankle flexors). However, complexity of that model, in which all hindlimb muscles were activated by both the CPG and somatosensory feedback and contributed to hindlimb energy generation and transfer, did not allow us to isolate and investigate in details the two major components of a whip-like motion. These components are (i) the generation of mechanical energy for motion by proximal hindlimb muscles and (ii) the energy transfer to the paw and creating the proximal-to-distal gradient of segmental angular velocities/accelerations by passive dynamics of distal muscles and body segments. Therefore, the goal of this study was twofold: (1) examine in intact cats if paw-shake mechanics correspond to the whip-like mechanism and (2) develop and analyze a simplified neuromechanical model of a cat hindlimb with a CPG, activating only hip muscles, and passive viscoelastic muscles of the knee, ankle, and metatarsophalangeal (MTP) joints that produce length/velocity-depended force. We hypothesized that cat paw shaking is organized as a whip-like motion and that specific muscle and inter-joint coordination in this motor behavior can emerge in interactions between a spinal CPG, hindlimb mechanical properties and muscle length/velocity-depended feedback.

## Materials and Methods

### Animal Experiments

All animal surgeries and chronic experiments were in compliance with the “Guide for the Care and Use of Laboratory Animals. Eighth Edition” (National Research Council of the National Academies, 2011) and were approved by the Institutional Animal Care and Use Committee of the Georgia Institute of Technology (protocol number A13063). Five adult female cats (mass 3.27 ± 0.55 kg, Table 1) participated in this and our previous studies and underwent previously described surgical and experimental procedures (Maas et al., 2010; Hodson-Tole et al., 2012; Mehta and Prilutsky, 2014; Gregor et al., 2018; Klishko et al., 2021). Briefly, the animals were trained to walk on a plexiglass enclosed walkway using food rewards. Major muscles of the right hindlimb were implanted with Teflon-insulated multistranded stainless-steel fine wires (CW5402; Cooner Wire, Chatsworth, CA) under sterile conditions and general isoflurane anesthesia. The animals recovered from surgery for 2 weeks with pain medication and antibiotics administered.

**TABLE 1:** Animal characteristics.

Cat	Mass, kg	Thigh length, mm	Shank length, mm	Tarsals length, mm	Digits length, mm	Paw-shake cycles analyzed
BL	3.00	100	100	77	31	23
BO	3.90	98	106	67	36	8
CO	3.83	95	119	66	30	2
JU	2.80	98	100	64	30	5
QL	2.80	93	103	59	30	3
Mean ± SD	3.27 ± 0.55	96.6 ± 2.7	105.5 ± 7.9	66.5 ± 6.6	31.4 ± 2.6	Total: 41

*Each animal is indicated by a 2-letter code. Recorded kinematics of these animals were used in the analysis of energy generation, transfer and absorption by joint forces and muscle moments. EMG recordings of cats BL, BO, CO and JU were also used in the analysis of EMG burst onset and offset times (see Table 2).*

We recorded kinematics of paw shaking using a 3D high-speed video motion-capture system (Vicon, United Kingdom) and reflective markers placed on the iliac crest, greater trochanter, knee joint, lateral malleolus, 5th metatarsophalangeal joint, and tip of 5th toe using double-sided adhesive tape. Prior to recordings, we attached a small piece of adhesive tape (2 × 3 cm) on the plantar surface of the right hindpaw and placed the cat inside the walkway. The cat walked across the walkway and periodically shook the hindlimb after initiating the swing phase; during paw shaking, the cat interrupted walking and was standing on the other three limbs. We recorded hindlimb kinematics and muscle electromyographic (EMG) activity at sampling rate of 120 and 3,000 Hz, respectively.

### Data Analysis

We used inverse dynamics analysis to compute resultant joint forces and joint (muscle) moments in the sagittal plane using recorded kinematics and inertial properties of cat hindlimb segments as described previously (Prilutsky et al., 2005; Farrell et al., 2014). Specifically, recorded vertical and horizontal marker displacements were low-pass filtered (Butterworth zero-lag filter, cut-off frequency 15 Hz). The recorded knee marker position was recalculated using measured lengths of the shank and thigh (Table 1) and recorded coordinates of the hip and ankle to reduce effects of skin movement around the knee (Goslow et al., 1973). We computed linear and angular velocities and accelerations of hindlimb segments using numerical differentiation. For further analysis, we selected only paw-shake episodes performed approximately in a sagittal plane (deviations of the thigh from the sagittal plane was within ∼25°). We then calculated the resultant joint forces and muscle moments using the computed accelerations and inertial properties of hindlimb segments; for review of inverse dynamics computations see, for example, Zatsiorsky (2002) and Winter (2004). The mass, position of the center of mass (COM) and moment of inertia with respect to COM of each hindlimb segment were calculated from the measured cat mass and body segment lengths (Table 1) using the regression equations developed in Hoy and Zernicke (1985).

**FIGURE 1:**
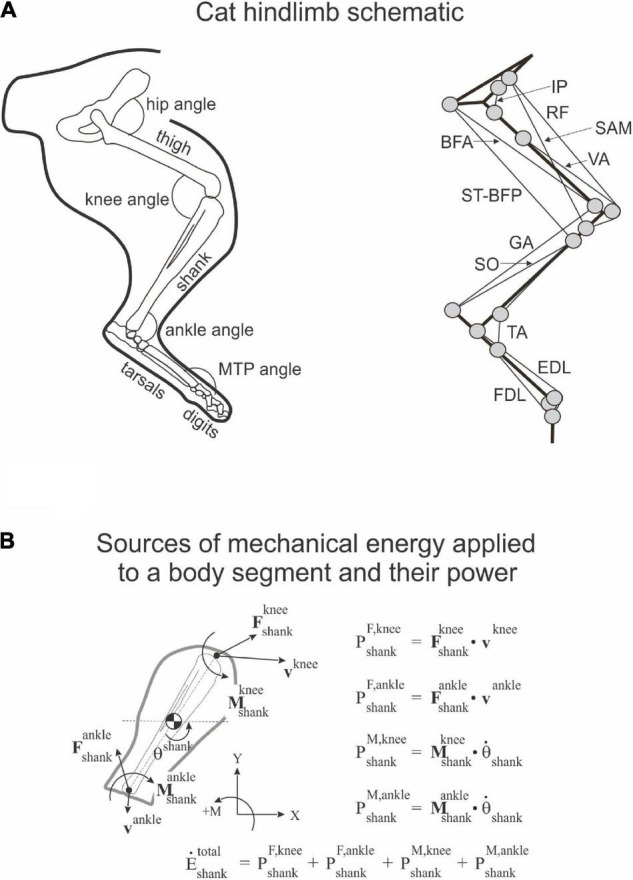
Schematic of cat hindlimb and sources of mechanical energy. **(A)** Cat hindlimb schematic. Left panel: Hindlimb segments and joint angles. MTP is metatarsophalangeal joint. Right panel: Schematic of the hindlimb musculoskeletal model. Thick and thin black lines denote limb segments and muscles, respectively; gray circles indicate locations of muscle origin, insertion and *via* points. IP, iliopsoas (hip flexor); BFA, biceps femoris anterior (hip extensor); SAM, sartorius medial (hip flexor and knee flexor); RF, rectus femoris (hip flexor and knee extensor); ST-BFP, semitendinosus-biceps femoris posterior (hip extensor and knee flexor); VA, vasti (knee extensor); GA, gastrocnemius (ankle extensor and knee flexor); SO, soleus (ankle extensor); TA, tibialis anterior (ankle flexor); EDL, extensor digitorum longus (flexor of digits; its ankle flexion action is neglected in the model); FDL, flexor digitorum longus (extensor of digits, its ankle extension action is neglected in the model). **(B)** Sources of mechanical energy applied to a body segment (shank) and their power (see section “Materials and Methods” and Eqs 1–4 for details).

To quantify energy generation and absorption by resultant muscle moments and energy transfer by resultant joint forces in the sagittal plane, we computed power produced by the resultant joint force and muscle moment at each hindlimb joint, as well as the rate of total energy change of each hindlimb segment (Robertson and Winter, 1980; see also Figure 1):

Rate of energy change of segment *s* due to power developed by joint force *F* at joint *j* (${\dot{E}}_{F,s,j}$):


(1) $${\dot{E}}_{F,s,j}=F_{x,s,j}v_{x,s,j}+F_{y,s,j}v_{y,s,j},$$

where *F*_*x,s,j*_ and *F*_*y,s,j*_ are two components of the force vector at joint *j* of segment *s*; *v*_*x,s,j*_ and *v*_*y,s,j*_ are two components of the linear velocity vector of joint *j* of segment *s*. Note that segments thigh, shank, and tarsals have a proximal and a distal joint (hip and knee for the thigh, knee and ankle for the shank, and ankle and MTP for the tarsals), while hindpaw (hind digits) has only the proximal MTP joint.

Rate of energy change of segment *s* due to power developed by muscle moment *M* at joint *j* (${\dot{E}}_{M,s,j}$):


(2) $${\dot{E}}_{M,s,j}=M_{s,j}\omega_{s},$$

where *M*_*s,j*_ is muscle moment at joint *j* of segment *s* and ω_*s*_ is angular velocity of segment *s*.

The rate of total energy change of segment *s* due to power of joint forces and muscle moments (${\dot{E}}_{s}$):


(3) $${\dot{E}}_{s}=\sum_{j}\left({\dot{E}}_{F,s,j}+{\dot{E}}_{M,s,j}\right).$$

The rate of total energy change of segment *s* computed as the time derivative of the total energy of segment *s* (${\dot{E}}_{s}^{\prime }$):


(4) $${\dot{E}}_{s}^{\prime }=\frac{d}{dt}\left(\frac{m_{s}v_{x,s}^{2}}{2}+\frac{m_{s}v_{y,s}^{2}}{2}+\frac{I_{s}\omega_{s}^{2}}{2}+m_{s}\text{g}y_{s}\right),$$

where *m_s_* and *I_s_* are segment mass and moment of inertia of segment *s* with respect to the segment COM, respectively; *v*_*x,s*_ and *v*_*y,s*_ are two components of the linear velocity vector of the COM of segment *s*; ω_*s*_ is the angular velocity of segment *s*; *y_s_* is vertical coordinate of the COM of segment *s*; and ***g*** is gravitational acceleration. Note that ${\dot{E}}_{s}={\dot{E}}_{s}^{\prime }$ (Robertson and Winter, 1980; Aleshinsky, 1986; Zatsiorsky, 2002).

**FIGURE 2:**
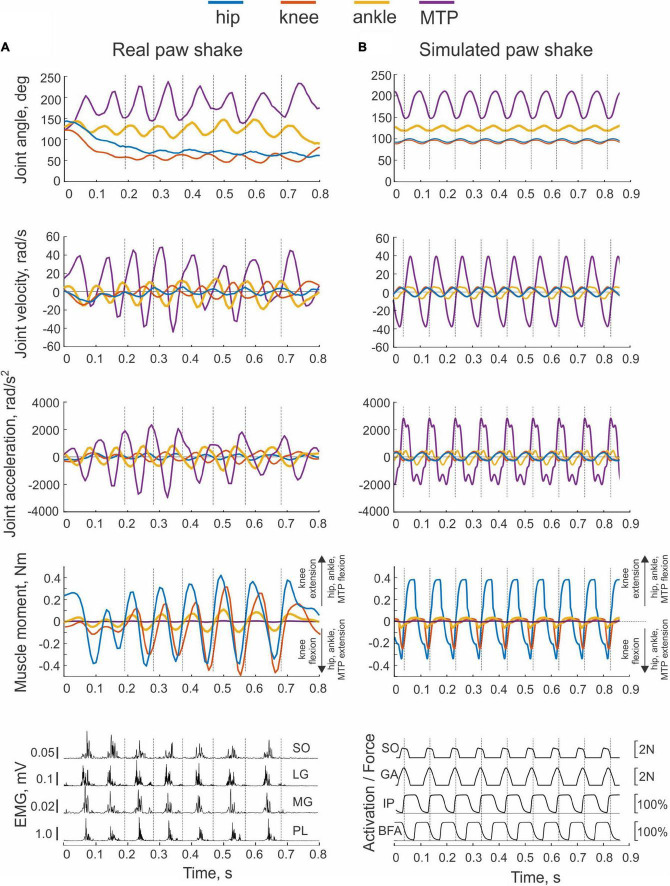
Examples of real and simulated paw shaking. Time periods separated by vertical dashed lines indicate cycles selected for analysis (cycle onset corresponds to the onset of the flexion hip moment). **(A)** A representative episode of real paw shaking of cat BL (Table 1). Panels from top to bottom: joints angles, joint angular velocities, joint accelerations, muscle moments, and EMG activities of ankle extensors SO (soleus), lateral gastrocnemius (LG), medial gastrocnemius (MG), and plantaris (PL). Positive muscle moment values correspond to flexion at the hip, ankle, and MTP joints, and to extension at the knee; negative moment values correspond to joint extension at the hip, ankle, and MTP joints, and to flexion at the knee. MTP is metatarsophalangeal joint. **(B)** Example of simulated paw shaking. The first 4 panels show the same mechanical variables as in **(A)**. Last bottom panel shows force produced by ankle extensors SO and GA, as well as activation of hip flexor IP and hip extensor BFA as % of maximal activation (see Figure 1 for muscle definition).

For these calculations, we used relatively steady-state paw-shake cycles (between 2 and 5) in the middle of each paw-shake episode [although there is a drift in the cycle duration (Smith et al., 1985; Parker et al., 2021)] and discarded the cycles in the beginning and the end. A cycle was defined as the period between two consecutive time onsets of a hip flexion moment (Figure 2).

To determine EMG burst onset and offset times during paw shaking, we analyzed EMG activity of 4 out of 5 cats from this study (BL, BO, CO, JU; Table 1) as well as EMG activity of additional 8 intact cats (Table 2) recorded in our previous study (Parker et al., 2021). We band-pass filtered (30–1,500 Hz), demeaned and full-wave rectified the recorded EMG signals. To reduce motion-depended noise, we slightly smoothed the rectified EMG signal using a zero-lag, moving average with an 8-ms window. We then determined EMG burst onset and offset times using 3 standard deviations (SD) from the EMG baseline and a condition of the minimum EMG burst duration of 25 ms. We defined the EMG baseline as the signal during a EMG inter-burst interval of at least 200 ms in duration. We determined the EMG burst onset and offset time of each muscle with respect to the EMG burst onset of the medial gastrocnemius muscle (GA) because this muscle was recorded in the largest number of paw-shake cycles.

**TABLE 2:** EMG burst onset and offset times of selected hindlimb muscles during a paw-shake cycle.

Cats	GA	SO		BFA		VA		TA		IP	
	Offset	Onset	Offset	Onset	Offset	Onset	Offset	Onset	Offset	Onset	Offset
BL	17.5 ± 1.2 / 6	1.9 ± 1.6 / 6	20.2 ± 2.2 / 6								
BO	45.4 ± 4.9 / 9	–3.3 ± 3.5 / 6	41.8 ± 7.0 / 6	7.2 / 3	29.0 / 3	13.0 / 1	27.0 / 1			31.5 ± 1.4 / 6	102.3 ± 13.6 / 6
BR	63.9 ± 10.0 / 24	–2.7 ± 4.4 / 21	48.3 ± 4.0 / 21			76.7 ± 16.4 / 5	127.1 ± 9.6 / 5			44.9 ± 7.5 / 15	117.2 ± 13.3 / 15
CO	38.3 / 3										
CR	56.4 ± 11.1 / 18	1.5 ± 8.4 / 15	40.9 ± 6.2 / 15	–1.1 ± 11.8 / 18	42.9 ± 13.6 / 18	80.7 ± 8.7 / 8	123.4 ± 5.8 / 8			28.5 ± 3.5 / 12	99.6 ± 8.7 / 12
JU	38.9 / 3	6.57 / 3	33.0 / 3					51.7 / 2	104.4 / 2	23.0 / 2	98.3 / 2
MO	51.4 ± 6.3 / 18							48.7 ± 4.1 / 12	102.0 ± 9.4 / 12	26.3 ± 7.2 / 5	88.9 ± 11.4 / 5
NA	62.4+11.4 / 6	2.7 ± 5.2 / 6	47.7+6.7 / 6	18.7 ± 5.9 / 6	33.5 ± 6.6 / 6						
SQ	79.2 / 3										
ST	42.1 ± 13.5 / 6	–20.3 / 3	45.6 / 3								
VE	53.8 ± 9.3 / 6					69.7 / 3	102.7 / 3	68.0 ± 3.8 / 6	149.2 ± 11.9 / 6		
WE	51.7 ± 11.4 / 21	–13.2 ± 6.9 / 18	43.8 ± 6.9 / 18							21.3 ± 4.61 / 6	123.9 ± 8.3 / 6
All	52.8 ± 14.6 / 123	–3.9 ± 9.0 / 78	42.4 ± 9.3 / 78	4.2 ± 13.0 / 27	39.3 ± 12.6 / 27	73.5 ± 19.1 / 17	115.2 ± 25.1 / 17	54.8 ± 9.7 / 20	116.5 ± 24.0 / 20	32.8 ± 10.4 / 46	107.6 ± 15.6 / 46

*Onset and offset measurements for all muscles were taken with respect to the EMG burst onset of the medial gastrocnemius muscle (GA) recorded in the same paw-shake cycles. The numerator shows the mean ± SD in ms; the denominator is the number of cycles included in the analysis. Standard deviations (SD) were not computed when the number of analyzed cycles was n ≤ 3. GA, gastrocnemius; SO, soleus; BFA, biceps femoris anterior; VA, vasti; TA, tibialis anterior; IP, iliopsoas. Independent factors muscle and cat significantly affected EMG burst onset and offset times [linear mixed model analysis; F(5, 275) = 284.1–393.6, p < 0.001 and F(11, 275) = 21.9–36.1, p < 0.001, respectively].*

### Neuromechanical Modeling of Paw Shaking

To simulate a whip-like motion of cat paw shaking, we simplified our previous neuromechanical model of the hindlimb with a half-center CPG and feedback signals (Bondy et al., 2016) developed using software for neuromechanical simulations AnimatLab (Cofer et al., 2010). We modeled hindlimb as a planar system of 5 rigid segments (pelvis fixed in place, thigh, shank, tarsals, and digits) that were interconnected by frictionless hinge joints and actuated by major hindlimb muscles crossing the hip, knee, ankle, and MTP joints (Figure 1A). Points of muscle origin and attachment, as well as the via points along the muscle paths, were selected to approximately match those described in the literature and to reproduce reported muscle moment arms with respect to the joints (Goslow et al., 1973; Prilutsky et al., 1996; Boyd and Ronsky, 1998; Burkholder and Nichols, 2004; MacFadden and Brown, 2007). Selected parameters of the hindlimb model segments and muscles are listed in Tables 3, 4, respectively. Hindlimb segments’ length and mass (Table 3) were selected to be within small ranges (5–20%) of the corresponding characteristics of the experimental cats (Table 1). Muscle parameters, including parameters of the three-element Hill-type muscle model (contractile element, parallel elastic element, series elastic element) were originally selected to match cat muscle properties reported in the literature (Goslow et al., 1977; Sacks and Roy, 1982; Herzog et al., 1992; Baratta et al., 1995; Scott and Loeb, 1995; Brown et al., 1996; Prilutsky et al., 1996). These muscle parameters included the optimal length of the muscle-tendon unit, parameters of the isometric force-length relationship, and muscle viscosity (the mean slope of the force-velocity relationship); resting length and stiffness of the parallel elastic element; stiffness of the series elastic element; and others (see Table 4 and AnimatLab model in Data Sheet 1 of Supplementary Material). These parameters were adjusted by hand within biologically realistic ranges to reproduce as close as possible joint angle patterns of real paw shaking (Figure 2).

**TABLE 3:** Hindlimb model segment parameters.

Segment	Length, mm	Mass, kg	Modified mass, kg
Thigh	92.7	0.146	0.006
Shank	98.6	0.059	0.019
Foot	59.3	0.019	0.059
Digits	25.0	0.006	0.146

*Length and mass of the model segments are within 5–10% of the values for the experimental cats, except the digits (20%) (see Table 1).*

**TABLE 4:** Hindlimb model muscle parameters.

Muscle	Maximum force, N	Optimal muscle-tendon length, mm	Muscle fiber length relative to the muscle-tendon unit length, %	Stiffness of parallel elastic element, kN/m	Stiffness of series elastic element, kN/m	Contractile element viscosity, Ns/m
IP	100	39.7	55	3.17	17.3	53.5
BFA	20	104.81	36	0.89	6.46	21.8
SAM	100	96.61	91	0.188	2.97	22.7
RF	100	93.0	20	0.5	50.0	197.5
ST-BFP	100	103.98	56	2.5	34.8	607.8
VA	100	94.58	27	7.9	37.3	534
GA	10	102.1	23	0.545	4.55	100
SO	10	74.65	58	0.062	0.99	100
TA	100	77.0	57	0.241	6.1	104
EDL	100	96.62	35	3.77	5.5	19.6
FDL	10	92.5	17	0.05	5.0	105

*These muscle parameters were first taken from the literature (Goslow et al., 1977; Sacks and Roy, 1982; Herzog et al., 1992; Baratta et al., 1995; Scott and Loeb, 1995; Brown et al., 1996; Prilutsky et al., 1996) and then adjusted by hand within biologically realistic ranges to reproduce joint angle patterns of paw shaking (see Figure 2). IP, iliopsoas (hip flexor); BFA, biceps femoris anterior (hip extensor); SAM, sartorius medial (hip and knee flexor); RF, rectus femoris (hip flexor and knee extensor); ST-BFP, semitendinosus-biceps femoris posterior (hip extensor, knee flexor); VA, vastus (knee extensor); GA, gastrocnemius (ankle extensor, knee flexor); SO, soleus (ankle extensor); TA, tibialis anterior (ankle flexor); EDL, extensor digitorum longus (ankle flexor, digits flexor); FDL, flexor digitorum longus (ankle extensor, digits extensor).*

In the simplified model, the half-center CPG provided rhythmic reciprocal excitatory inputs to motoneurons of one-joint hip flexor iliopsoas (IL) and one-joint hip extensor biceps femoris anterior (BFA). The other more distal hindlimb muscles in the model were considered passive and did not receive any excitatory or inhibitory inputs. The model was actuated by active contractions of the hip flexor and extensor muscles. Stiffness coefficients of the parallel and series elastic components, as well as a viscosity coefficient of the contractile component of the passive muscles allowed them to produce length- and stretch velocity-depended force. This model design was implemented to simulate a whip-like mechanism of movement organization more precisely. In this model, mechanical energy for motion is supplied only by most proximal hip muscles, whereas the other more distal muscles can only dissipate or return some of this energy to the system.

The model of a half-center CPG was described in detail elsewhere (Bondy et al., 2016; Parker et al., 2018). Briefly, each half-center was a spiking Hodgkin-Huxley neuron with six ionic currents. The half-centers interacted through an inhibitory synaptic current. The low voltage of half-inactivation and large time constant of inactivation of this current prevent large calcium currents during high frequency (∼10 Hz) paw-shake-like rhythmic activities. Parameters of this CPG model generating paw-shake-like activity can be found in Bondy et al. (2016) and Parker et al. (2018).

We used simulated kinematics and kinetics of paw shaking generated in AnimatLab forward simulations to compute power of joint forces and muscle moments, as well as the rate of total energy change as described by Eqs (1)–(4). We also investigated effects of mass distribution along the hindlimb on the proximal-to-distal gradient of segmental angular velocities and accelerations. The mass and cross-section gradient of a real whip is critical for developing an extremely high velocity of the whip tip (Krehl et al., 1998; McMillen and Goriely, 2003). To this end, we reversed the gradient of segmental masses in the hindlimb model (Table 3) and ran AnimatLab forward simulations using the same CPG input and muscle properties. In addition, we conducted simulations in which we changed muscle stiffness parameters (Table 4) by ± 20% from the nominal values and kept all other model parameters unchanged. This relatively small range of muscle stiffness changes led to a large increase in the joint angle movement magnitudes exceeding in some joints the physiologically feasible ranges (e.g., the knee joint was slightly overextended). Similar simulations were performed to investigate the role of muscle viscosity after changing it by ± 90%.

### Statistics

We tested effects of hindlimb segments and joints (independent factors) on experimentally determined peaks of joint and segmental angular velocity and acceleration, muscle moments, and segmental energy changes due to joint forces and muscle moments (depended variables). We used a linear mixed-effects model (MIXED) with paw shaking cycle as a random factor (IBM SPSS Statistics 27, Chicago). We used a similar linear-mixed model to test effects of independent factors muscle and cat on the EMG burst onset and offset time, as well as on the EMG burst duration in a cycle (depended variables). Pairwise comparisons were performed using Bonferroni adjustments. The significance level was set at 0.05.

## Results

### Real Paw Shaking in the Cat

#### General Characteristics

Kinematic variables of paw shaking of a single cat shown in Figure 2A were representative of all 5 cats. The cycle of steady-state paw shaking averaged across all cycles and animals was 0.098 ± 0.014 s, which corresponded approximately to 10 Hz. The range of joint angles and peak joint velocities increased from proximal to distal joints. For example, mean peaks of MTP and ankle flexion velocities were 34.2 ± 16.3 rad/s and 12.9 ± 3.7 rad/s, respectively, while the peak of hip flexion velocity was 3.1 ± 1.8 rad/s (joint angles are defined in Figure 1A). The effect of joint as an independent factor on the peaks of flexion joint velocities was significant; [*F*(3, 144) = 57.297, *p* < 0.001]. Similar trends occurred for peaks of extension velocities in these joints (25.3 ± 11.1 rad/s, 12.1 ± 2.9 rad/s and 3.1 ± 2.4, for MTP, ankle and hip joints, respectively; [*F*(3, 144) = 80.128, *p* < 0.001]. Peaks of joint moments had the opposite tendency—higher peaks occurred in proximal joints (Figure 2A). For example, on average across all cats and cycles, peaks of flexion MTP and ankle moments (0.005 ± 0.002 Nm and 0.073 ± 0.018 Nm) were significantly lower of the peak hip flexion moment [0.292 ± 0.091 Nm; *F*(3, 144) = 209.4, *p* < 0.001]. Note that in the first half of the paw-shake cycle, a combination of MTP flexion, ankle flexion, knee extension and hip flexion joint moments occurred, whereas in the second half, these moments changed direction to MTP extension, ankle extension, knee flexion and hip extension (Figure 2A). Activity of distal muscles started when the joint was moving in the direction opposite to the muscle action. For example, ankle extensors (soleus, lateral and medial gastrocnemius and plantaris) demonstrated EMG burst in the middle of each cycle when the ankle was flexing (negative ankle angular velocity; Figure 2A).

**FIGURE 3:**
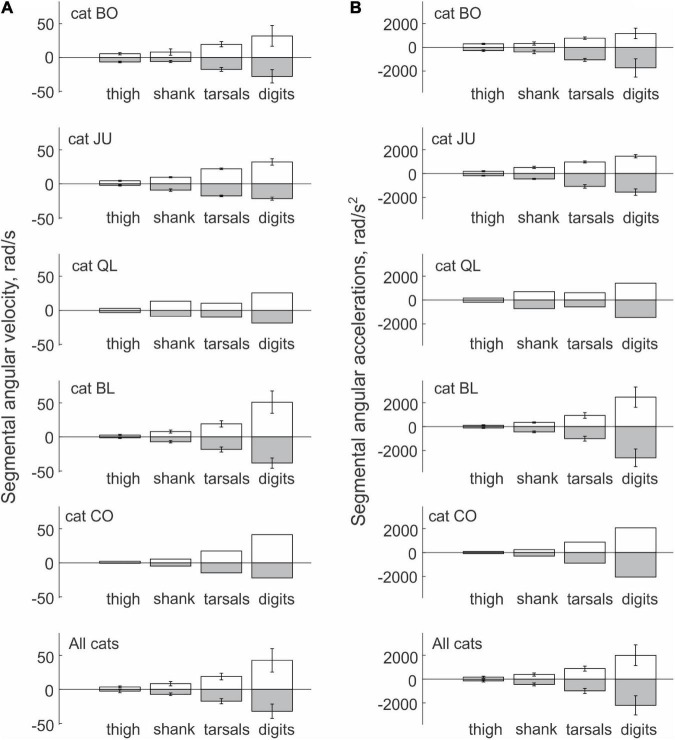
Mean (± SD) peak values of segmental angular velocity **(A)** and acceleration **(B)** computed across individual paw-shake cycles of individual cats and all cats together. Standard deviations (SD) for cats QL and CO were not computed because of the small number of cycles analyzed (see Table 1 for the number of analyzed cycles).

All 5 cats demonstrated a proximal-to-distal gradient of segmental angular velocity and acceleration peaks in both flexion and extension directions during paw shaking (Figure 3). The mean peak of flexion velocity and acceleration across all cats and cycles increased from 3.491 ± 1.595 rad/s and 152.5 ± 84.2 rad/s^2^ for the thigh to 42.6 ± 17.1 rad/s and 1992.2 ± 874.5 rad/s^2^ for the digits (paw) [see Figure 3 and Data Sheet 2 in Supplementary Material; *F*(3, 144) = 71.4–71.9, *p* < 0.001]. The corresponding peaks of extension angular velocity and acceleration were 2.635 ± 2.237 rad/s and 161.7 ± 79.3 rad/s^2^ for the thigh to 32.1 ± 10.5 rad/s and 2200.9 ± 807.4 rad/s^2^ for the digits [Figure 3; *F*(3, 144) = 92.4–120.3, *p* < 0.001].

#### Energy Generation and Transfer by Muscle Moments and Joint Forces in Real Paw Shaking

**FIGURE 4:**
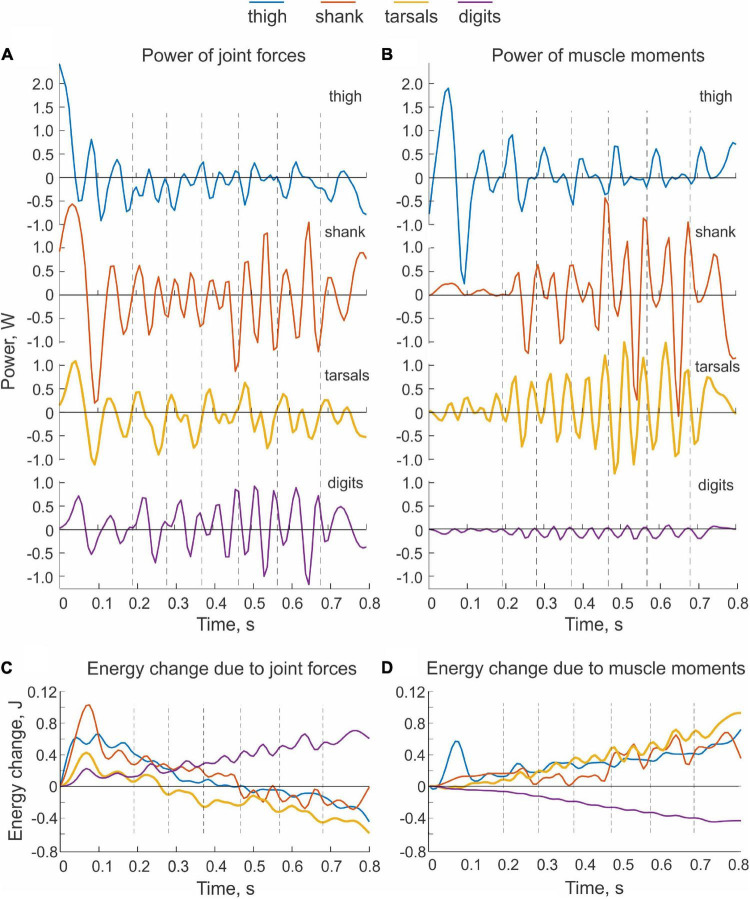
Examples of power of sources of mechanical energy applied to hindlimb segments and the corresponding changes in total energy of the segments during real paw shaking. Cat BL. **(A)** Total power of joint forces applied to each body segment. **(B)** Total power of muscle moments applied to each body segment. **(C)** Changes of total energy of each body segment due to the action of joint forces. Energy changes were computed by integration of the corresponding total power **(A)** over time. **(D)** Changes of total energy of each body segment due to the action of muscle moments. Energy changes were computed by integration of the corresponding total power **(B)** over time.

Typical patterns of power of joint forces and muscle moments applied to each hindlimb segment are shown in Figures 4A,B, respectively. Power of forces at the thigh joints during steady-state paw shaking had mostly negative values, indicating that the thigh energy was reduced by the joint forces applied to this segment. The hip and knee joint forces decelerated the thigh, which is confirmed by the decrease of thigh energy obtained by integration of thigh power due to joint forces over time (Figure 4C). The energy decrease in the thigh due to the actions of the hip and knee forces corresponds to energy transferred to the neighboring segments, including the shank, by these forces. However, total power of joint forces applied to the shank was more negative than positive (Figure 4A) as confirmed by the decrease of the shank energy over the whole paw-shake episode due to the action of the knee and ankle joint forces applied to the shank (Figure 4C). This means that energy transferred from the thigh to the shank was further transferred to the tarsals by the ankle joint force. Although the tarsals receive energy from the shank, energy of tarsals decreased (Figure 4C) due to its transfer by the MTP joint force to the digits, whose energy during paw shaking increased (Figure 4C). Energy transferred from the thigh, shank and tarsals to the digits was generated by muscle moments at the hip, knee and tarsals, which is evident from mostly positive power of muscle moments acting on the thigh, shank and tarsals (Figure 4B) and from growing energy of these segments over the paw shaking episode (Figure 4D). Power of the muscle moment applied to the digits is negative (Figure 4B), i.e., muscles at the MTP joint absorb and dissipate energy of the digits.

**FIGURE 5:**
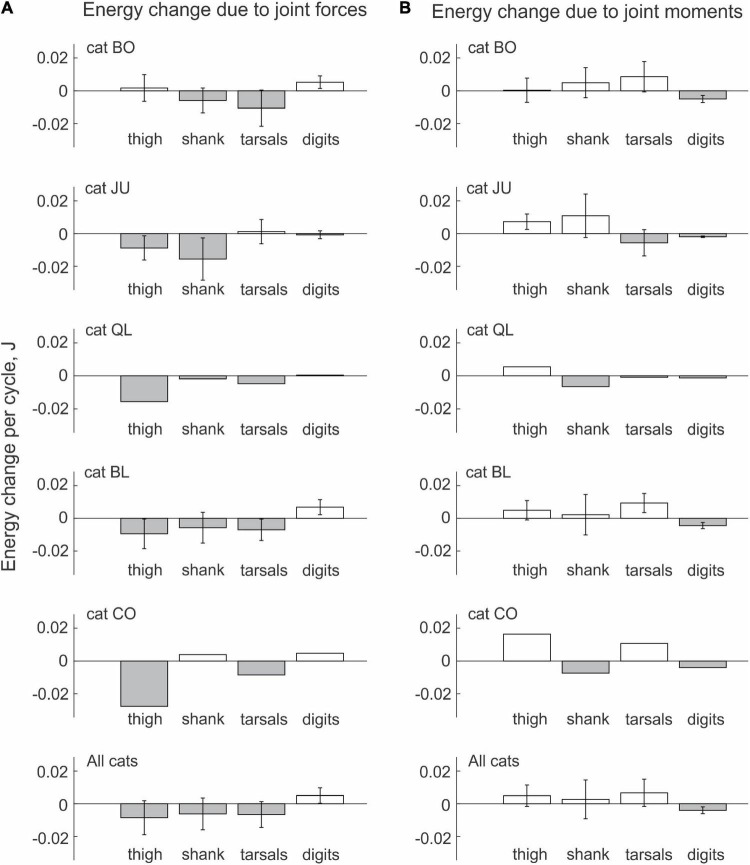
Mean (± SD) energy change of each hindlimb segment per cycle of real paw shaking due to the action of joint forces **(A)** and muscle moments **(B)** computed across paw-shake cycles of individual cats and all cats together. Standard deviations (SD) for cats QL and CO were not computed because of the small number of cycles analyzed (see Table 1 for the number of analyzed cycles).

We observed similar patterns of energy generation, absorption and transfer in all cats. The energy change of the thigh, shank and tarsals in each paw-shake cycle due to the action of joint forces was negative in most cases (Figure 5A). The digits received energy (positive energy change) through joint forces in 3 out 5 cats, while two cats showed essentially no change in energy on average. On average across all cats and cycles (Table 1), the thigh, shank and tarsals lost energy due to the action of joint forces—the corresponding values were 8.5 ± 0.4 mJ, 6.2 ± 9.7 mJ and 6.6 ± 7.9 mJ (Figure 5A and Data Sheet 2 in Supplementary Material). The joint force at the MTP increased energy of the digits, 5.0 ± 4.7 mJ. The effect of the segment factor on the energy transfer was significant [*F*(3, 144) = 14.3, *p* < 0.001] with significant differences among all segments (*p* < 0.011), except between the shank and tarsals (*p* = 0.696). Mostly opposite trends of segmental energy change were caused by muscle moments (Figure 5B). Muscle moments increased energy of the most proximal segments, i.e., the thigh (in 4 cats), shank (in 2 cats) and tarsals (in 3 cats); energy of the most distal segment, the digits, was absorbed by muscle moments in all 5 cats. On average across all cats and cycles, the energy changes in the thigh, shank, tarsals and digits were 4.9 ± 6.6 mJ, 2.7 ± 11.8 mJ, 6.7 ± 8.3 mJ and –4.0 ± 2.0 mJ (Figure 5B). The effect of the segment factor on these energy changes was significant [*F*(3, 144) = 7.698, *p* < 0.001]. Pairwise comparisons revealed significant differences in energy change between the digits and thigh (*p* < 0.001), the digits and tarsals (*p* = 0.001), but not between the digits and shank (*p* = 0.069). The energy change of the shank was significantly lower than that of the thigh (*p* = 0.008), but was not different from that of the tarsals (*p* = 0.114). The energy change of the thigh and tarsals were not statistically different either (*p* = 0.280).

**FIGURE 6:**
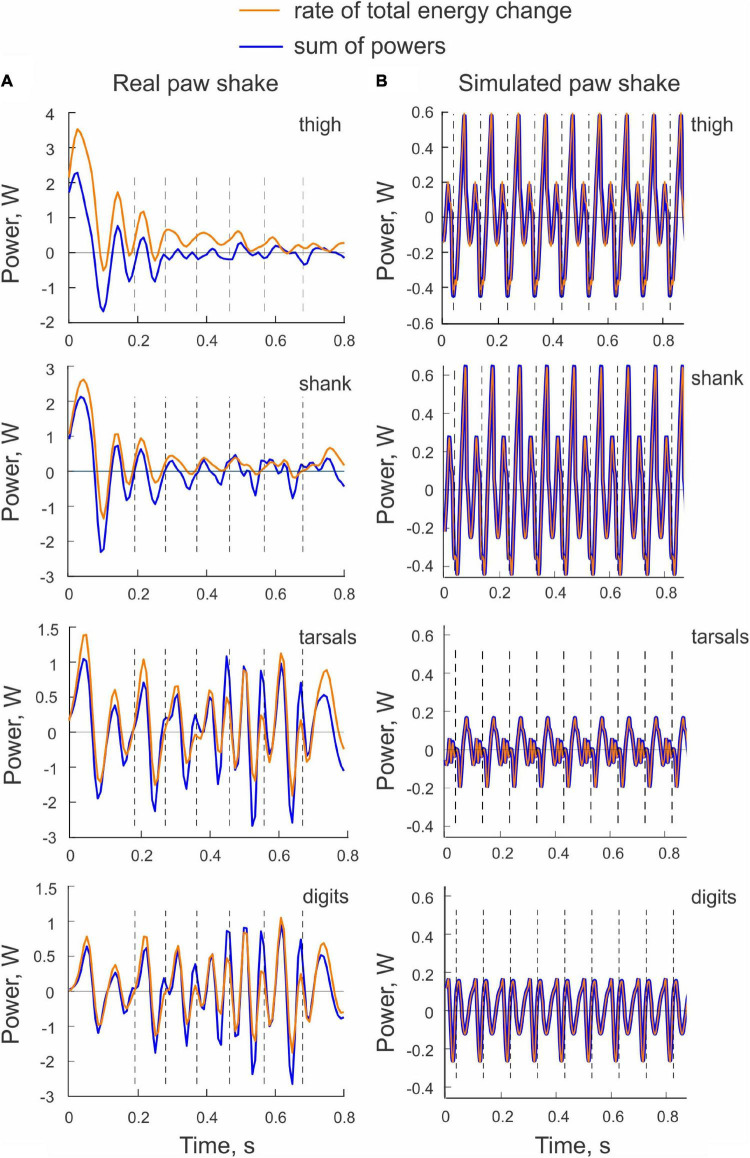
Examples of the rate of total energy change and the sum of powers of all sources of energy for each hindlimb segment computed for a real paw shaking episode of cat BL **(A)** and for simulated paw shaking **(B)**.

The sum of powers of all joint forces and muscle moments applied to a body segment (${\dot{E}}_{s}$, Eq. 3) should be equal to the rate of change of the total energy of the segment (${\dot{E}}_{s}^{\prime }$, Eq. 4). This was not the case in the example shown in Figure 6A, although the similarity of the two patterns was apparent. Possible reasons for the observed differences will be considered in Discussion.

### Simulated Paw Shaking

#### General Characteristics of Simulated Paw Shaking

**FIGURE 7:**
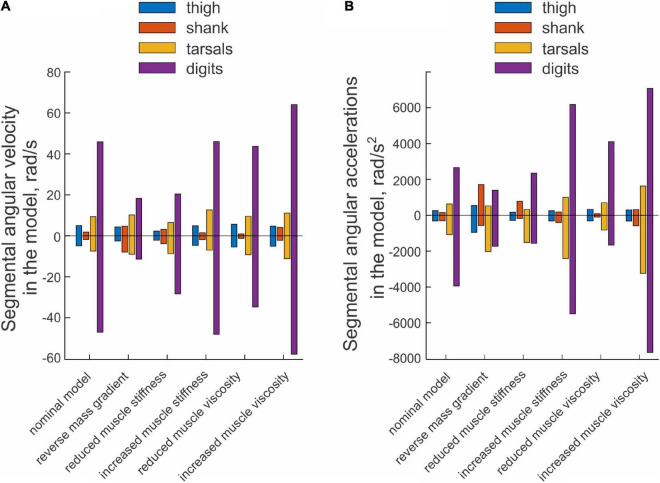
Mean peaks of segmental angular velocity **(A)** and acceleration **(B)** computed across 10 simulated paw-shake cycles for different versions of the model. Nominal parameters of the model are listed in Tables 3, 4. In model with reverse mass gradient, segmental masses were changed so that mass of hindlimb segments decreased in the distal-to-proximal direction (see Table 2). In model with reduced and increased muscle stiffness, stiffness of muscle parallel and series elements was reduced and increased by 20% from the nominal values, respectively (see Table 4). In model with reduced and increased muscle viscosity, viscocity of muscle contractile element was reduced and increased from the nominal values by 90%, respectively (see Table 4).

An example of paw shake simulation results is shown in Figure 2B. The ranges of joint motion and the peaks of joint velocities were smaller than during real paw shaking for the knee and ankle and similar for the hip and MPT joints. Correspondingly, the hip flexion and extension moments and the knee flexion moment had peak magnitudes similar to those in real paw shaking. The model produced a very small knee extension moment (below 0.03 Nm). The simulated ankle flexion and extension moments were much lower than observed in the experiments. There were differences in joint movement phases between simulated and experimental paw shaking. Although simulated muscle moments demonstrated the experimentally observed muscle moment combinations in the first and second half of the cycle (hip flexion-knee extension-ankle flexion and hip extension-knee flexion-ankle extension, respectively), some phase shifts of angular displacements and velocities between joints in simulated paw shaking were different compared to the real one. For example, the peak of MTP flexion angle coincided with the peak of ankle extension angle in simulations (Figure 2B), whereas these events were separated by about 25% of the cycle time in real paw shaking (Figure 2A). In addition, changes in hip extension and flexion angles coincided perfectly with changes in knee flexion and extension angles in simulations, whereas in real paw shaking these angle changes were shifted in time by a half of the cycle. Nevertheless, the model demonstrated a clear proximal-to-distal gradient of the segmental angular velocities and accelerations with peak values for the hip and MPT joints closely matching the experimental values (compare Figures 7A,B, nominal model with Figures 3A,B, all cats). The simulated muscle moment peaks likewise demonstrated the distal-to-proximal gradient (compare Figure 2B with Figure 2A).

#### Energy Generation and Transfer by Muscle Moments and Joint Forces in Simulated Paw Shaking

**FIGURE 8:**
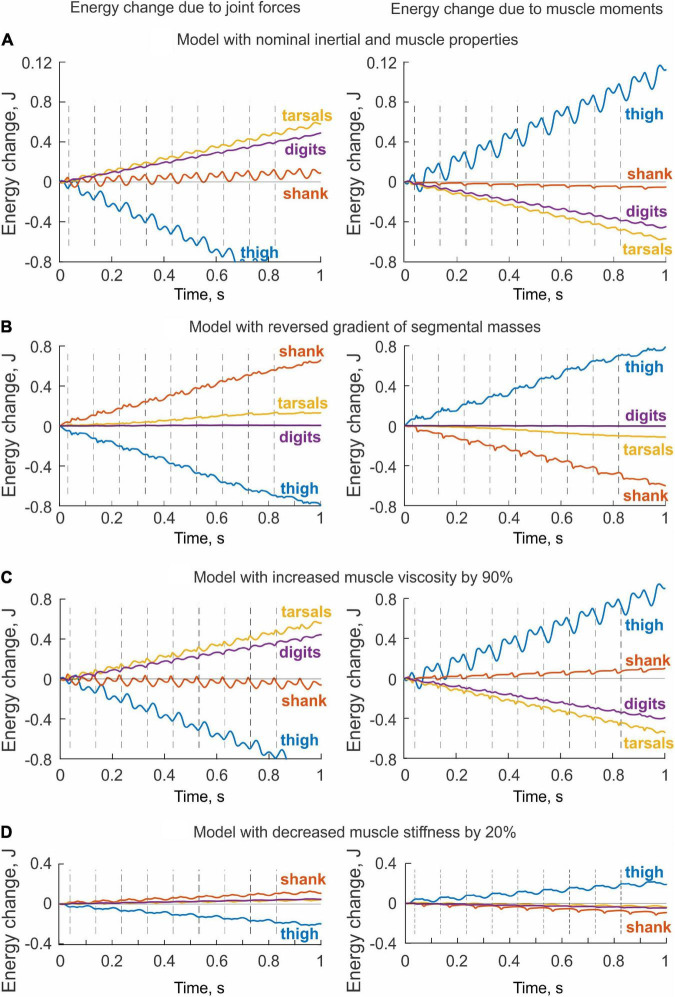
Changes of total energy of each body segment due to the action of joint forces (left panels) and muscle moments (right panels) computed for simulated paw shaking of different versions of the model. **(A)** Model with nominal inertial and muscle properties. **(B)** Model with reverse gradient of segmental masses (Table 3). **(C)** Model with increased muscle viscosity by 90% (Table 4). **(D)** Model with decreased muscle stiffness by 20% (Table 4).

Since the hindlimb model was designed to generate energy by one-joint hip muscles only, we expected that muscle moments would increase energy of the thigh only and that this energy would be transmitted to the shank by the knee joint force, that is, the thigh would lose energy through the action of the joint forces applied to the thigh. This is exactly what we observed (Figure 8A). Energy delivered to the thigh by muscle moments increased throughout the simulated paw shake episode and matched approximately the thigh energy loss due to the action of the knee joint force (power of the hip joint force was zero because the hip was fixed in place)—compare energy values in the left and right panels at 0.7 s in Figure 8A. All other muscles of the hindlimb model were passive and thus could not generate energy by themselves. However, these muscles produced passive forces as a function of muscle length and stretch velocity. As seen in Figure 8A (right panel), muscle moments applied to the shank, tarsals and digits absorbed energy and thus decreased energy of these segments. The joint forces applied to the shank, tarsals and digits increase their energy (Figure 8A, left panel). In fact, the total energy gain of these segments corresponded to the energy loss of the thigh through the action of the joint forces since the muscle moments did not generate energy. The increase of shank energy was relatively small because most of energy received by the shank from the thigh was delivered to the tarsals through the ankle joint force. The tarsals in turn transferred about half of energy received from the shank to the digits (Figure 8A, left panel).

**FIGURE 9:**
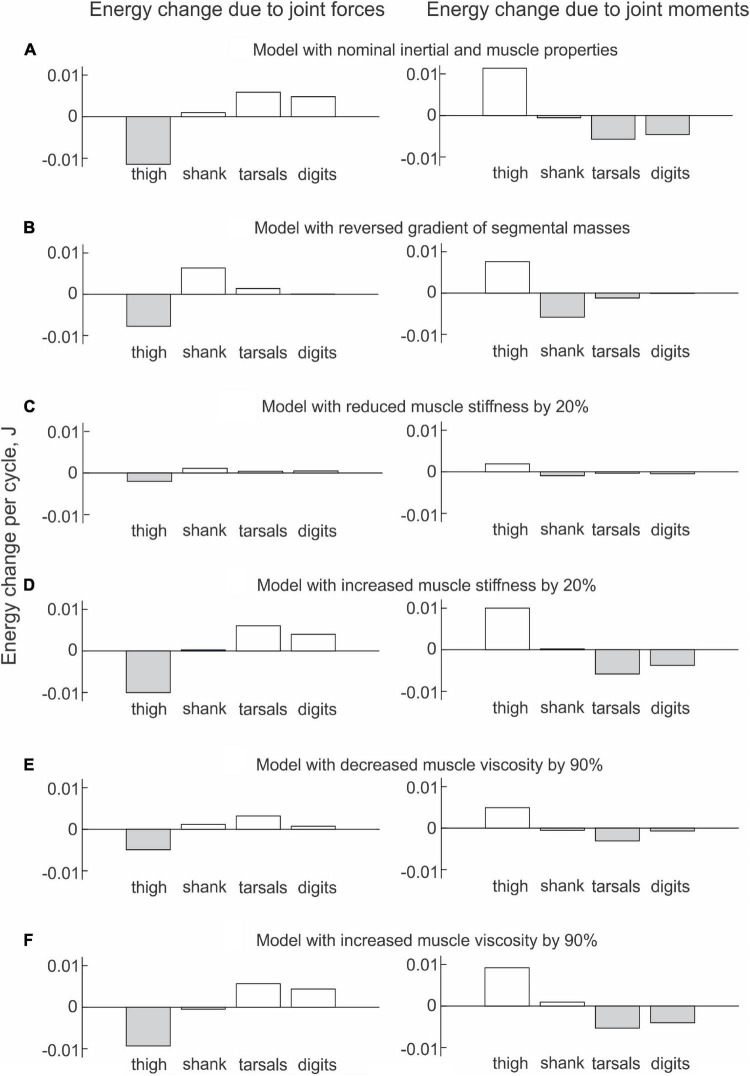
Mean energy change of each hindlimb segment per cycle of simulated paw shaking due to the action of joint forces (left panels) and muscle moments (right panels) computed across 10 cycles of paw-shake simulations performed for different versions of the model. **(A)** Model with nominal inertial and muscle properties. **(B)** Model with reversed gradient of segmental masses. **(C)** Model with reduced stiffness of the parallel and series elastic elements of each muscle by 20%. **(D)** Model with increased stiffness of the parallel and series elastic elements of each muscle by 20%. **(E)** Model with reduced viscosity of the contractile element of each muscle by 90%. **(F)** Model with increased viscosity of the contractile element of each muscle by 90%.

The energy changes of hindlimb segments per cycle of simulated paw shaking likewise show that energy of the thigh was increased by muscle moments at the hip (11.5 mJ), while muscle moments decreased energy of the more distal tarsals (−5.7 mJ) and digits (−4.6 mJ); energy of the shank did not change substantially (–0.5 mJ; Figure 9A, right panel). Energy loss in the thigh and energy gain in the tarsals and digits due to joint forces were opposite to the energy gain and loss due to muscle moments. Correspondingly, energy of the shank increased little (1.0 mJ) because almost all energy it received from the thigh (11.4 mJ) was transferred to the tarsals (5.9 mJ) and digits (4.8 mJ); Figure 9A, left panel; see also Data Sheet 2 in Supplementary Material.

Comparisons between the sum of all powers delivered to each segment by the joint forces and muscle moments (${\dot{E}}_{s}$, Eq. 3) and the rate of change of the total energy of each segment (${\dot{E}}_{s}^{\prime }$, Eq. 4) demonstrated a perfect match (Figure 6B). This verifies the correctness of our segmental energy analysis.

#### Effects of Hindlimb Inertia Distribution and Muscle Viscoelastic Properties on Simulated Energy Transfer

The change in hindlimb mass distribution altered energy exchange among hindlimb segments. Energy generated by muscle moments applied to the thigh (Figure 8B, right panel) was transferred by the joint knee moment to the shank (the decrease in the thigh energy due to the knee joint force led to the increase in shank energy; Figure 8B, left panel). However, little energy was transferred from the shank to the tarsals and especially to the digits. For example, the digits received only 0.059 mJ of energy in the paw shake cycle out of 7.8 mJ transferred from the thigh (Figure 9B, left panel). The tarsals received slightly more energy per cycle (1.381 mJ; Figures 8B, 9B, left panels). As a result, there was a substantial decrease in peaks of angular velocity (by 3–4 times) and acceleration (by over 2 times) of the digits compared to simulations with the nominal segmental masses, and the proximal-to-distal gradient almost disappeared, especially for accelerations (Figure 7).

We also investigated the effect of muscle elastic properties (stiffness of the parallel and series elastic elements, Table 4) on the whip-like mechanism in the model because elasticity was shown to play a role in transmission of whip waves (McMillen and Goriely, 2003) and because in our model, distal hindlimb muscles develop passive length-dependent forces. We changed the nominal stiffness values of all muscles in the model by ± 20% and conducted simulations with the same neural inputs to motoneurons of hip muscles IP and BFA. The decrease in stiffness increased the joint angle magnitudes and led to a slight overextension at the knee by about 20°. Simulations with increased muscle stiffness demonstrated anatomically realistic ranges of motion, i.e., without knee overextension. We did not increase the range of stiffness changes further to keep the changes symmetric and simulations anatomically realistic. Examples of segmental energy changes with decreased muscle stiffness are shown in Figure 8D). Energy delivered to the thigh by muscle moments during simulated paw shaking were much smaller than in the model with nominal stiffness. This was caused by smaller tendon forces due to much more compliant tendon of the hip muscles (not shown). Energy transfer from the thigh to the distal segments were minimal (2 mJ; Figures 8D, 9C, left panels) due to low forces of the distal muscles. The changes in energy generation and transfer with reduced stiffness were also reflected in a reduced angular velocity and acceleration of the digits compared to the nominal model (Figure 7). The increase in muscle stiffness by 20% resulted in paw shake simulations similar to those of the nominal model. Specifically, there was comparable supply and absorption of segmental energy per cycle by the muscle moments and energy transfer from the thigh to the tarsals and digits by the joint forces in the two simulations (Figure 9D). In addition, the two simulations had comparable proximal-to-distal gradients of segmental angular velocities and accelerations (Figure 7).

Since muscle force development and energy absorption in the model depend on muscle viscosity, we also investigated effects of muscle viscosity on the whip-like mechanism. The model was less sensitive to changes in muscle viscosity, so we conducted simulations with viscosity changed by ± 90% from its nominal values in all muscles (Table 4). Segmental energy changes in simulated paw shaking with increased muscle viscosity by 90% (Figure 8C) were comparable to the changes in the model with nominal parameters (Figure 8A). For example, the difference in energy changes due to joint forces in the thigh, shank, tarsals and digits were 2.1 mJ (18% of the nominal model), 0.51 mJ (52%), 0.20 mJ (3%), and 0.43 mJ (9%), respectively. The corresponding values for segmental energy changes due to joint moments were 2.15 mJ (19%), 0.42 mJ (44%), 0.40 mJ (7%), and 0.53 mJ (12%), respectively. One noticeable difference was that muscle moments supplied small amount of energy to the shank (the change of energy per cycle is positive compared with a negative change in the nominal simulations; compare Figure 8A with Figure 8C, Figure 9A with Figure 9F). This was caused by a slightly greater stretch velocity-depended muscle moments (quantified as the mean of moment peaks) of the knee extensors (0.035 Nm vs. 0.026 Nm) and flexors (–0.302 Nm vs. –0.249 Nm). Energy transferred by the joint forces from the thigh and shank to the tarsals and digits was about the same as in the nominal model (Figures 8C, 9F). The proximal-to-distal gradient of segmental velocities and accelerations was preserved in this case with higher digits velocity and acceleration than in the nominal model (Figure 7). The decrease in muscle viscosity by 90% resulted in very small muscle forces, in paw shaking that was very different from the natural one, and in small energy generation, absorption and transfer per cycle (Figure 9E). The velocity and acceleration gradients were, however, generally preserved (Figure 7).

#### Muscle Coordination in Simulated Paw Shaking

**FIGURE 10:**
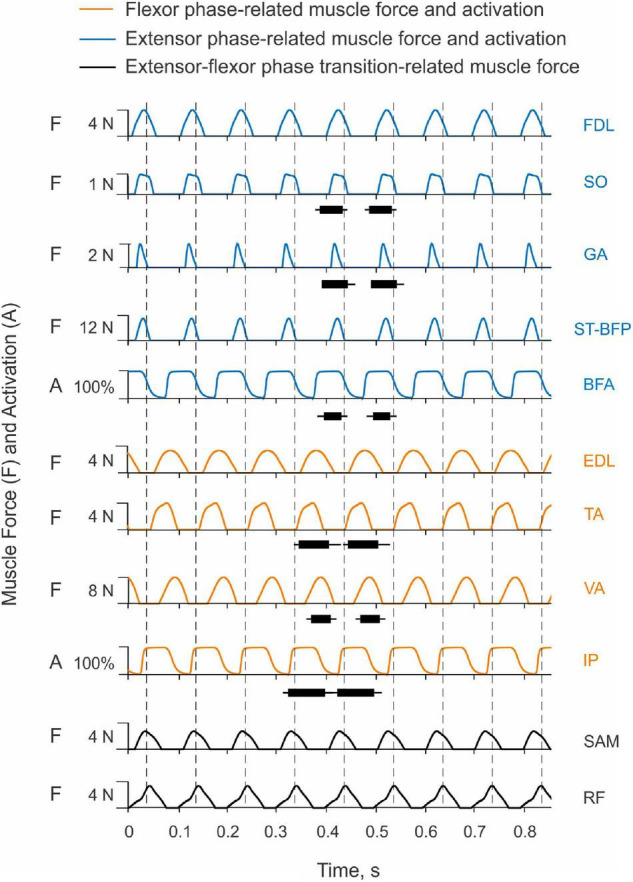
Activation patterns of one-joint hip muscles and passive motion-depended forces of more distal hindlimb muscles during simulated paw shaking. One-joint hip flexor iliopsoas (IP) and one-joint hip extensor biceps femoris anterior (BFA) receive excitatory input from the CPG in the flexor and extensor phases, respectively; their activation (A) is shown in % of maximum activation. Passive motion-depended forces of the remaining muscles (F) are shown in N. Vertical dashed lines separate paw-shake cycles. The cycle onset is defined as the onset of the hip flexion moment (see Figure 2). The EMG burst durations for selected muscles are shown for 2 cycles as horizontal bars connecting the EMG burst onset and offset times measured in real paw shaking (mean ± SD, Table 2). To compare the simulated muscle activation and forces with the measured EMG burst durations, IP EMG burst onset was aligned with the IP simulated activation onset in a cycle. The horizontal black bars shown in two cycles are identical. For muscle abbreviation (see Figure 1 and Table 4).

Only two muscles in the model received excitatory input from the CPG, hip flexor IP and hip extensor BFA. Their simulated activation was reciprocal with little overlap at the cycle onset (the extensor-flexor phase transition at the onset of hip flexion moment) and at mid-cycle (the flexor-extensor phase transition at the onset of hip extensor moment; Figure 10). Onset of the simulated hip flexion moment (paw shake cycle onset, indicated by vertical dashed lines in Figure 10) occurred with a delay of 14 ms after onset of simulated IP activation. Onset of the hip extension moment occurred in the middle of the cycle, 16 ms after BFA activation onset. By model design (see section “Materials and Methods”), the other hindlimb muscles did not receive neural input. They produced passive viscoelastic force (Figure 10) as a function of the muscle length and stretch velocity (not shown but can be inferred from the joint angles and velocities in Figure 2B). For example, the ankle flexor TA developed passive force in the first half of the cycle (Figure 10) when the hip moment is flexion and ankle is extending (Figure 2B), i.e., when TA is lengthening. The MTP flexor EDL likewise produced force in the first half of the cycle, although its force production lasted slightly longer since the maximal length of this muscle (or MTP extension angle) in the cycle was reached slightly later than in TA (the TA peak length occurs at peak of the ankle extension; Figure 2B). The knee extensor VA produced force almost in phase with the MPT flexor EDL and ankle flexor TA, although the onset of VA force production was delayed compared to all other flexors (by 10–17 ms, Figure 10). The VA passive force production coincided with the knee flexion phase (Figure 2B), during which VA was lengthening. In simulations, the hip extensor BFA was activated in the second half of the paw-shake cycle (Figure 10). The force development of the MTP and ankle extensors (FDL, SO, GA) and of a two-joint hip extensor-knee flexor ST-BFP occurred in the last 30–40% of the cycle (Figure 10) in phase with lengthening of these muscles (or extension of the knee and flexion of the ankle and MTP joints; Figure 2B). Proximal two-joint muscles SAM (hip and knee flexor) and RF (hip flexor and knee extensor) produced their peak forces close to the extensor-flexor phase transition (Figure 10) when the hip and knee joints were extending and their angular velocities reached the maximum values (Figure 2B).

Despite simplicity of the hindlimb neuromechanical model, in which only hip one-joint muscles produced active muscle force and generated mechanical energy while the other muscles produced passive length- and stretch velocity-depended forces, the time of simulated force development was generally consistent with periods of EMG bursts recorded in selected muscles during real paw shaking. For example, anterior hindlimb muscles that accelerate the hindlimb forward (flexors IP, TA, EDL and extensor VA) produced force in the first half of the cycle when the EMG bursts of IP, TA and VA occurred in real paw shaking (Figure 10 and Table 2). Ankle extensors (SO and GA) and hip extensor BFA produced their EMG bursts in the second half of the cycle in phase with the simulated forces or activation of these muscles. These muscles also demonstrated reciprocal EMG activity with and shorter EMG burst durations than the corresponding ankle and hip flexors (TA and IP; Figure 10 and Table 2). Specifically, the EMG burst durations of extensors SO, GA and BFA were 46.3 ± 13.9 ms, 52.8 ± 14.6 ms and 35.1 ± 17.1 ms, respectively. These values were significantly shorter than the EMG burst durations of flexors TA (61.7 ± 16.5 ms) and IP (74.8 ± 16.1 ms); [*F*(5, 305) = 33.8, *p* < 0.014]. The durations of the simulated force development by extensors FDL, SO, GA and ST-BFP and flexors EDL and TA had similar trends (24 ms–38 ms vs. 55 ms–69 ms, respectively, Figure 10). Interestingly, the knee extensor VA demonstrated a greater co-activation with flexors TA and IP (EMG burst overlaps were 42 and 34 ms, respectively) than with extensors SO, GA and BFA (overlap between 8 and 12 ms), resembling the corresponding simulated force patterns (Figure 10).

## Discussion

### Study Goals and Hypotheses

One goal of the study was to examine if paw shaking in cats is organized as a whip-like movement, in which energy generated by larger proximal muscles is transferred to distal segments by joint forces; for review see Zatsiorsky (2002). This whip-like mechanism has been suggested to contribute to developing high velocities of the distal segments of the arm and leg during fast throwing and kicking movements in humans (Putnam, 1991; Hirashima et al., 2003) and knee extension during the swing phase of locomotion in cats and humans (Chapman and Caldwell, 1983; Martin and Cavanagh, 1990; Wisleder et al., 1990). We confirmed and expended to the digits previous reports (Hoy et al., 1985; Hoy and Zernicke, 1986) demonstrating the proximal-to-distal gradient of segmental angular velocities and accelerations during cat paw shaking. We showed for the first time that during paw shaking, energy delivered to hindlimb segments proximal to the digits by muscle moments was transferred to the digits by the joint forces.

The second goal was to develop a simplified neuromechanical model of a cat hindlimb with a half-center CPG, activating hip flexor and extensor muscles, and passive viscoelastic distal muscles. We also investigated if this model could reproduce whip-like paw shake movements, i.e., the proximal-to-distal gradient of segmental angular velocities and accelerations, as well as energy generation and absorption by muscle moments and energy transfer in the proximal-to-distal direction by the joint forces. We found that this model was able to reproduce the above features of the whip-like motion. In addition, we demonstrated that the mass distribution along the hindlimb and muscle stiffness had major effects on the generation of high angular velocity and acceleration of the digits during paw shaking and on the transfer of energy to the digits.

### Whip-Like Mechanism

The term whip-like motion has been used in biomechanics and neural control of movement to describe motion of multi-segmented open kinematic chains of upper and lower extremities, the goal of which is to develop a high linear or angular velocity of the last segment of the kinematic chain; see for example (Zatsiorsky, 2002). The progressively increasing angular velocity of more distal segments in this motion has been explained in part by the action of joint force applied at the proximal joint of distal segments that creates a moment of force with respect to the center of mass of the distal segment and increases its the angular acceleration and velocity (Chapman and Caldwell, 1983; Martin and Cavanagh, 1990; Putnam, 1993; Zatsiorsky, 2002).

Experimental studies and theoretical analyses of propagation of whip waves along real whips demonstrated that the reduction of the cross-sectional area and mass of the whip in the proximal-to-distal direction is an important factor in increasing the whip tip velocity above the speed barrier and achieving translational accelerations of 50,000 times greater than the acceleration of gravity (Krehl et al., 1998; McMillen and Goriely, 2003). This result is explained by the laws of conservation of energy and momentum. The energy and momentum imparted to the whip at the handle travels as a whip wave along the whip, and the decrease in mass along the whip must increase the speed of the traveling wave, given constancy of the momentum. Although the mechanism of velocity enhancement along a real whip is likely different from the whip-like mechanism of energy transfer in kinematic chains, these two mechanisms appear to shear some common features. Efficiency of energy transfer and enhancement of velocity in the whip and in the cat hindlimb model depends on mass distribution along the length of the two systems and on their elastic properties.

The cat hindlimb inertia and muscle mass and volume also decrease in the proximal-to-distal direction (Sacks and Roy, 1982; Hoy and Zernicke, 1985), as reflected in mass of body segments of our model (Table 3). This mass distribution in the hindlimb not only enhances angular velocity and acceleration of the digits in our simulated paw shaking, it also decreases hindlimb inertia with respect to the hip joint and thus reduces energy expenditure during the swing phase of locomotion (e.g., Martin, 1985). This cat hindlimb inertia and muscle volume distribution is also observed in the forelimbs of cats (Hoy and Zernicke, 1985; Martin et al., 2010) and in human arms (Zatsiorsky, 2002; Holzbaur et al., 2007). The greater ability of larger proximal muscles to generate mechanical energy for movement may also explain a typical organization of arm reaching movements. Largest muscles around proximal (leading) joints generate muscle moments that drive the arm toward the target, while muscle moments at the distal (trailing) joints counter the interaction motion-dependent moments (Sainburg et al., 1999; Dounskaia, 2010). This joint coordination strategy was suggested to reduce the number of control variables, simplify neural control of reaching and minimize neural effort for joint coordination (Dounskaia and Shimansky, 2016).

Our paw-shake simulations with changed muscle viscoelasticity of distal hindlimb muscles suggested an important role of this muscle property in muscle and inter-joint coordination and efficient energy transfer along the hindlimb. This result is consistent with previous studies demonstrating the importance of limb stiffness regulation in various postural and movement tasks (Mussa-Ivaldi et al., 1985; Nichols et al., 1999; Frolov et al., 2006). This regulation is primarily mediated by muscle length and force-depended spinal reflexes (Eccles et al., 1957a,b; Fritz et al., 1989; Nichols, 2018). The length and stretch-velocity sensitive muscle spindle group Ia afferents from the triceps surae and hamstrings muscles demonstrate extremely high firing rates during cat paw shaking (Prochazka et al., 1989). This afferent activity occurs in phase with stretch and EMG burst of these muscles, indicating that these muscles absorb energy of the flexing tarsals and extending shank and slow them down, as we observed in our simulations (Figures 8A, 9A). In real paw shaking, we observed energy absorption by muscle moments of only the most distal segment, the tarsals (Figures 4D, 5B). This is because energy received by the shank and tarsals from the action of joint forces and muscle moments exceeded energy transported from them to the digits. Thus, it appears that length- and stretch velocity-depended feedback from distal segments help regulate the proper timing of muscle activity to coordinate inter-joint coordination that allows for efficient energy transfer and absorption. The role of this motion-depended feedback in distal muscles of our simulation model played their passive viscoelastic properties, i.e., the dependence of muscle force on muscle length and stretch velocity. In fact, timing of forces produced by the passive muscles during simulated paw shaking were remarkably similar to the periods of EMG bursts of selected muscles during real paw shaking in this (Figures 2A, 10) and other studies (Smith et al., 1985). This similarity includes the atypical co-activation between ankle and hip flexors TA and IP and knee extensor VA. In the model, the VA force generation is caused by VA elongation during limb forward acceleration due to activation of IP. During real paw shaking, length-depended feedback from elongating VA can also contribute to the VA EMG burst in phase with flexors, although other factors including central commands are also likely to contribute. EMG bursts of VA during real paw shaking is more variable than in other muscles, with bursts missing occasionally or consisting of two parts—in phase with flexors and in phase with extensors (see Figure 3 in Smith et al., 1985). Double activity bursts in the VA nerve spanning the flexor and extensor phases were also reported in fictive paw-shake-like activity, i.e., without motion-depended feedback (Pearson and Rossignol, 1991). This suggests that central inputs from spinal CPG contribute to EMG activity of VA in real paw shaking. CPG is likely to contribute substantially to EMG activity of other hindlimb flexors and extensors, since they produce activity bursts in the corresponding flexor and extensor phases defined by the direction of the flexion and extension muscle moments (Figures 2A, 10). Additional evidence for substantial contributions of central inputs to the EMG magnitude of ankle extensors was provided in Mehta and Prilutsky (2014); in that study removal of stretch reflex from these muscles by muscle self-reinnervation had no effect on their EMG burst timing and relative magnitude.

### Possible Organization of Neural Control of Paw Shaking

Since joint kinematics and kinetics, inter-joint coordination, and EMG activity patterns (with co-activation of TA and VA) of cat paw shaking appeared drastically different from those of cat locomotion (Hoy et al., 1985; Smith et al., 1985, 1998; Carter and Smith, 1986; Hoy and Zernicke, 1986; Koshland and Smith, 1989; Prochazka et al., 1989), it has been suggested that paw shaking is controlled by a substantially reconfigured locomotor unit-burst CPG (Carter and Smith, 1986). In addition, motion-depended sensory feedback was suggested to affect primarily EMG activity of the knee extensor VA and ankle flexor TA, but not other muscles, because hindlimb deafferentation or limb casting led to changes in their EMG burst onsets and offsets (Smith and Zernicke, 1987; Koshland and Smith, 1989). The organization of the mammalian CPG controlling rhythmic behaviors, such as different forms of locomotion, scratching, and paw shaking, is not fully understood. Although many researchers agree that some common elements of the CPG network can be used to control different rhythmic movements, there is an ongoing debate about whether the spinal CPG has a single-level or a multi-level architecture (McCrea and Rybak, 2008; McLean and Dougherty, 2015; Ausborn et al., 2021; Grillner and Kozlov, 2021; Klishko et al., 2021). In the former, unit-bursts generators do not receive common flexor- and extensor-related rhythmic inputs and can be flexibly reorganized by sensory and/or central inputs to meet mechanical demands of various motor behaviors (Grillner, 1981; Carter and Smith, 1986; Grillner and Kozlov, 2021). In the latter, a top half-center CPG layer sets a common rhythm and phase for all flexor and extensor last order interneurons controlling motoneuronal activities of flexors and extensors, and a lower CPG layer can adjust the duration and magnitude of flexor and extensor activity of the corresponding motoneurons based on sensory and/or central inputs without necessarily changing the rhythm, the so-called non-resetting effects (McCrea and Rybak, 2008). Our previous study (Bondy et al., 2016) demonstrated that even a classic single-level half-center CPG organization (Brown, 1911, 1914) in combination with specifically organized motion-dependent feedback could reasonably predict basic features of mechanics and muscle activity of walking and paw shaking. The current study extended previous results to demonstrate that a simple half-center CPG, controlling activity of hip flexors and extensors, with autogenic length and stretch-velocity feedback controlling force production in more distal muscles, can provide a proper inter-joint and muscle coordination for energy generation and transfer to the digits and for providing the proximal-to-distal gradient of segmental angular velocities and accelerations during paw shaking. The proper coordination appears to emerge from interactions between the half-center CPG, inertial properties of hindlimb segments, and muscle length and stretch-velocity feedback. The neural control of paw shaking seems well adjusted to the natural passive dynamics of the hindlimb and thus requires minimal intervention. Similar well-adjusted interactions between natural dynamics of the musculoskeletal system and neural control have been proposed based on demonstrations of human-like walking in a passive physical model (McGeer, 1990) and swimming of a dead fish against the flow (Beal et al., 2006). Future studies will need to reconcile the current and previous findings suggesting the importance of length feedback for regulation of activity of ankle and knee muscles during paw shaking (Koshland and Smith, 1989; Prochazka et al., 1989; Bondy et al., 2016) with the fact that removal of monosynaptic length feedback from ankle extensors by muscle self-reinnervation does not affect their activity patterns in paw shaking (Mehta and Prilutsky, 2014).

### Limitations of the Study

We noticed substantial differences between the rate of the total energy of hindlimb segments (Eq. 4) and the sum of powers of the joint forces and muscle moments applied to these segments (Eq. 3); Figure 6A. These mechanical variables must be identical in accordance with the law of conservation of energy. Our inverse dynamics analysis and computer code were correct since we used the same code to compute these variables for the model and obtained a perfect match (Figure 6B). The differences between the real and simulated paw shaking are that the latter is strictly planar and produced by a model composed of ideal rigid segments interconnected by frictionless hinge joints with fixed location and orientation of joint axes. To perform inverse dynamics analysis of real paw shaking we assumed constant segment lengths and inertial properties and constant position and orientation of the rotation axis at each joint. These assumptions seem justified for the cat as the body COM acceleration computed from kinematics of a walking cat was similar to the COM acceleration obtained from the recorded ground reaction forces (Manter, 1938). However, real paw shaking was not fully planar. As explained in Methods, we discarded paw shakes in which the thigh was abducted-adducted by more than ∼25°. Still, in the retained cycles, there were instantaneous deviations from the sagittal plane, which could potentially explain the observed differences between the rate of total energy changes and the total power for the segments, although the exact reason for this offset is unclear. Another source of observed differences, especially in the ranges between maximal and minimal values of the two variables, could be small random errors in digitized marker coordinates magnified by the numerical differentiation, e.g., Winter (2004). This explanation is consistent with greater peak values of the total segmental total power, which requires more numerical differentiation (e.g., computing second time derivatives of linear and angular accelerations for obtaining joint forces and muscle moments) than for computations of the rate of energy change. Given the similar proximal-to-distal direction of energy flow in the real and simulated paw shakes (Figures 4, 5, 8A, 9A), the described discrepancies do not appear to affect our major conclusions.

Another limitation of the study was that our paw-shake simulations did not fully match the real paw shaking. The largest discrepancies of simulated paw shaking were in the in-phase changes of knee and hip angles and velocities and in the small knee extension moment (Figure 2). We expected that our simplified version of a more complex previous model (Bondy et al., 2016) would not reproduce precisely all aspects of real paw shaking. In the simplified model, we removed CPG input to all but two hip muscles and eliminated muscle spindle length-depended excitation to all hindlimb muscle motoneurons, except for IP and BFA. The force production in the passive viscoelastic distal muscles of this model depended on muscle length and stretch velocity. This was done to make the hindlimb model and paw-shake simulations more similar to a whip and to the process of energy generation at the proximal end of the whip and to the velocity enhancement along the whip length. Despite the mentioned discrepancies, the model clearly reproduced the energy generation, transfer and absorption (compare Figures 4C,D, 5 with Figures 8A, 9A), the proximal-to-distal gradient of segmental angular velocity and acceleration (compare Figure 3 with Figure 7), and timing of muscle force production in selected muscles (Figures 2, 10).

## Data Availability Statement

The original contributions presented in the study are included in the article/Supplementary Material, further inquiries can be directed to the corresponding author.

## Ethics Statement

The animal study was reviewed and approved by the Institutional Animal Care and Use Committee of Georgia Institute of Technology.

## Author Contributions

BP conceived and designed the study, carried out the experiments, data analysis and model development, and drafted the manuscript. JP helped with data analysis and model development, and critically revised the manuscript. GC helped with conceiving and designing the study, carried out model development, and critically revised the manuscript. AK helped with conceiving and designing the study, carried out the experiments, data analysis and model development, and critically revised the manuscript. All authors contributed to the article and approved the submitted version.

## Conflict of Interest

The authors declare that the research was conducted in the absence of any commercial or financial relationships that could be construed as a potential conflict of interest.

## Publisher’s Note

All claims expressed in this article are solely those of the authors and do not necessarily represent those of their affiliated organizations, or those of the publisher, the editors and the reviewers. Any product that may be evaluated in this article, or claim that may be made by its manufacturer, is not guaranteed or endorsed by the publisher.
